# ASSESSMENT OF KNOWLEDGE AND SELF EFFICACY BEFORE AND AFTER TEACHING BASIC LIFE SUPPORT TO SCHOOLCHILDREN

**DOI:** 10.1590/1984-0462/2021/39/2019143

**Published:** 2020-08-03

**Authors:** Maria de Lurdes Rovisco Branquinho Pais Monteiro, Ana Isabel Borges Ferraz, Fernanda Maria Pereira Rodrigues

**Affiliations:** a Centro Hospitalar e Universitário de Coimbra, Portugal.

**Keywords:** Learning, Education, Cardiopulmonary resuscitation, Child, Aprendizagem, Educação, Reanimação cardiopulmonar, Criança

## Abstract

**Objective:**

Teaching basic life support to schoolchildren is well established as one of the most effective strategies in increasing bystander CPR rates. However, there is a lack of scientific evidence concerning the Portuguese pediatric population. The present study aims to evaluate the outcome of a basic life support training session on theoretical knowledge and self-efficacy, immediately after the training and 6 months later, in a pediatric population.

**Methods:**

A total of 392 schoolchildren, aged seven to 12 years old, participated in this prospective longitudinal study, answering a questionnaire before, immediately after, and six months after receiving 120 minutes of resuscitation training from medical students.

**Results:**

There was a significant increase in the knowledge and self-efficacy after one single training session. Both decreased over a period of six months but remained significantly higher than the baseline. These results were homogeneous across classes.

**Conclusions:**

Medical students provided adequate basic life support training to a group of Portuguese schoolchildren, with effects in the knowledge and self-efficacy lasting for at least six months.

## RESUMO

**Objetivo:**

O ensino de suporte básico de vida a crianças em idade escolar está bem estabelecido como uma das estratégias mais eficazes no aumento das taxas de reanimação cardiorrespiratória. No entanto, há uma falta de validação científica para a população pediátrica portuguesa. Este estudo pretende avaliar o resultado de uma sessão de treino em suporte básico de vida no conhecimento teórico e autoeficácia, imediatamente e seis meses após, em população pediátrica.

**Métodos:**

Um total de 392 crianças, com idade entre 7-12 anos, participaram neste estudo longitudinal prospetivo, respondendo a um questionário antes, imediatamente depois e 6 meses após receberem 120 minutos de treino em reanimação cardiorrespiratória feito por estudantes de medicina.

**Resultados:**

Houve um aumento significativo no conhecimento e autoeficácia nas crianças após este treino único. Ambos diminuíram num período de seis meses, mas mantiveram-se significativamente mais altos que o valor basal. Estes resultados foram homogêneos nas diferentes turmas.

**Conclusões:**

Estudantes de medicina ministraram adequadamente o treino de Suporte Básico de Vida a um grupo de crianças portuguesas em idade escolar, com efeitos em nível do conhecimento e da autoeficácia que duram pelo menos seis meses.

## INTRODUCTION

Out-of-hospital cardiac arrest (OHCA) is a major public health problem, responsible for a significant number of deaths in Europe.^1^ The importance of shortening the treatment-free interval after cardiac arrest is well established, since the survival rate from OHCA increases two to four times with early initiation of cardiopulmonary resuscitation (CPR).^2^ Thus, bystander CPR is crucial as it can be promptly initiated before the arrival of an Emergency Medical Service, reducing the treatment-free interval after the collapse.^3^ However, in many European countries, the current bystander resuscitation rates are lower than 30%,^4^ and it is estimated that doubling that rate could triple the chance of survival in those situations.^5^

As bystanders with previous CPR training are more likely to perform CPR,^6^ training the population arises as a strategy with promising results in increasing survival following OHCA.^5^ The motivation to start CPR is highly dependent not only on the level of knowledge but also on the level of confidence in the ability to perform it correctly, both of which tend to increase after a hands-on training session.^7^ ^,^ ^8^

Although there are several strategies to increase lay CPR rates recommended in the European Resuscitation Council (ERC) Guidelines for Resuscitation 2015,^9^ training schoolchildren in CPR has been demonstrated to be feasible, one of the most effective^8^ ^,^ ^10^ and with more sustained results when it starts at younger ages (under 12 years old).^11^

In order to promote schoolchildren’s education in CPR worldwide, the European Patient Safety Foundation (EuPSF), the ERC, the International Liaison Committee on Resuscitation (ILCOR) and the World Federation of Societies of Anesthesiologists (WFSA) developed the “Kids Save Lives” position statement on schoolchildren’s education in CPR, which asserts that “teaching CPR to all schoolchildren will lead to a marked improvement in global health”.^12^ In 2015, this was endorsed by the World Health Organization (WHO).^13^

Portugal is part of a group of countries that has legislation about CPR education (Resolução da Assembleia da República n.º 33/2013. Diário de República. 1.ª Série. 53. 2013-03-15). However, legislation is not enough and must be supported by effective implementation and surveillance strategies.^14^ ^,^ ^15^ Additionally, there is still a lack of scientific evidence about the best way to do it, particularly in the Portuguese pediatric population.^16^

Following the “Kids Save Lives” recommendations,^12^ the Medical Student Nucleus of the Coimbra Academic Association (NEM/AAC) developed a project entitled *A Brincar*, *A Brincar*, with medical students teaching Basic Life Support (BLS) to schoolchildren. As part of this project, the present study aims to evaluate the outcome of a single 120-minute BLS training session on theoretical knowledge and self-efficacy related to performing BLS, immediately after the session, and 6 months later. Another goal of this study is to evaluate whether different tutors lead to different outcomes in terms of knowledge and self-efficacy, since training sessions were taught to each class by a different pair of medical students.

## METHOD

This six-month prospective longitudinal study was performed between November 2016 and June 2017; it included 392 schoolchildren, aged seven to 12 years old, in ten public and two private Portuguese elementary schools, located in Coimbra and Viseu. It was a convenience sample, limited by the geographical area where the medical students could possibly go, which included a group of schools that agreed to participate. Students with special learning needs were also included.

The schoolchildren answered a baseline questionnaire before the training session. The same questionnaire was applied one day to one week after the training session, and then six months later. Pupils who attended the training course but didn’t complete the baseline questionnaire were excluded from the study. However, students who answered the baseline questionnaire but didn’t answer the second or third questionnaires were included.

The CPR trainers were 84 medical students from the University of Coimbra. They all underwent a 90-min theoretical training course, which included a BLS update based on the 2015 ERC Guidelines for Resuscitation,^17^ as well as some basic skills for communication with children. The instructors were two Pediatricians of the Hospital Pediátrico de Coimbra.

Each 2-hour session occurred during school time and assembled two trainers and 12 to 25 students. All training sessions were standardized, using the same support presentation, which was made based on the 2015 ERC Guidelines for Resuscitation,^17^ with the guidance of elementary school teachers. It included an age-appropriate lecture on BLS, as well as hands-on training on CPR and recovery position (RP). The concepts were presented in a problem-based learning setting, which allowed not only to further engage the children, but also to give them practical examples of the application of that knowledge.

As it was not possible to use mannequins because of the lack of funding, the hands-on training on compressions was highly limited. In order to overcome that flaw, each schoolchild used a stuffed animal in order to perform the correct technic regarding the positioning of the hands, arms and torso, as well as the rate of the compressions. The trainers corrected each child as they were performing the compression technic, while listening to a song about the BLS basics, with a beat of 100 beats per minute. The RP was performed in pairs, step by step, with the help of one tutor for each pair.

The theoretical knowledge of the children about CPR was tested using a 10-item questionnaire, available upon request from the corresponding author, with eight multiple choice questions and two ordering questions. A literature review failed to identify a validated questionnaire that could be used in this study. The questionnaire used was built with the guidance of elementary school teachers and Educational Specialists. Each multiple-choice question was made to evaluate one of the key messages that were selected as the most relevant information. Two ordering questions were also included, regarding the BLS algorithm and the RP, the latter using the images showed in the presentation. Although other similar studies included questions regarding theoretical knowledge on the biology of circulation and ventilation,^8^ ^,^ ^11^ ^,^ ^18^ those were not included, as it was assumed that all children in the study would have been assessed for that knowledge in previous years, since it was part of their curricular program.^19^

The content of the questionnaire was validated through a pre-test with 36 schoolchildren who did not receive the BLS training session, from one private and one public schools and in the same education grade class as the population in the study, to ensure that each item was understandable and the answers unambiguous to children in that age group.

Points for correct answers were added up and then divided by the maximum score (10/10). The overall score is thus presented in the form of a percentage, meaning that the higher the percentage, the better the schoolchildren’s state of CPR knowledge.

Self-efficacy was used to evaluate the schoolchildren’s confidence in their own ability to perform CPR. It was measured using a four-item questionnaire, available upon request from the corresponding author, adapted with the author’s permission from one used in another study^8^ performed in Germany, over a six years period, in children aged ten to 16 years old. The statements were translated from English to Portuguese, and the four-point scale was adapted to a simpler two-point scale (yes *versus* no), since it has been concluded in the pre-test that the children had some trouble finding the difference between “fairly true”, “true” and “strongly true”. Points for either “yes” (sentences one, two and four) or “no” (sentence three) were added up to provide an overall score from zero points (low confidence) to four points (high confidence).

The statistical analysis was carried out using IBM *Statistical Package for the Social Sciences* (SPSS) for Macintosh, version 24.0 (IBM Corp., Armonk, NY, USA). Categorical data are described by absolute numbers and percentages; continuous data are described by means, standard deviations, maximum and minimum. The effectiveness of the training courses in terms of knowledge and self-efficacy was tested using general linear models with repeated measurements. Statistical significance was considered as a p<0.05.

The study received approval (CE-083/2017) from the Ethics Committee of the Faculty of Medicine of University of Coimbra, Coimbra, Portugal on September 25, 2017.

The participation was voluntary, and each child was informed that they could withdraw at any time. Parents gave written consent before the beginning of the study. The communication with the parents was done through the teachers, who fully supported the project from the start.

## RESULTS

**Figure 1:**
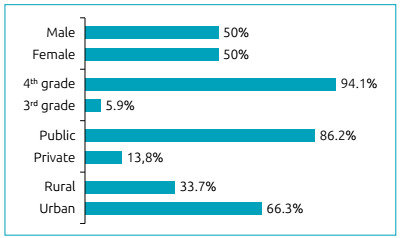
Demographic data.

The study included a total of 392 schoolchildren with an age ranging from seven to 12 years, mean±standard deviation (SD) of 8.9±0.6 years, and a ratio of boys to girls of 1:1. The students were from 12 different schools and 21 different classes; 94.1% of them were fourth graders and 86.2% were from public schools. The 21 classes had between 11 to 24 children participating in the study, with a mean±SD of 18.7±4.0 students each. Demographic data are presented in Figure 1. Of the 392 students that answered the baseline questionnaire, 372 (94.8%) answered the second and 376 (95.9%) the third questionnaires.

A one-way repeated measured analysis of variance (ANOVA) was conducted to evaluate the null hypothesis that there was no change in schoolchildren’s knowledge on BLS when measured before, one day to one week, and six months after the participation in a single 2-hour BLS training course (n=392). Descriptive statistics are presented in Table 1. The results of ANOVA indicated a significant time effect (Wilks’ Lambda=0.2, F(2,359)=690.8, p<0.05, η^2^=0.8), with significant evidence to reject the null hypothesis. The Mauchly’s Test of Sphericity indicated that the assumption of sphericity had not been violated, chi-square(2)=0.5, p=0.78.

**Table 1:** Percentage of correct answers in the questionnaire assessing the theoretical knowledge: descriptive statistics.

	Mean	Standard deviation
Baseline	37.3	11.5
One day to one week after training	71.8	16.4
6-months after training	60.6	15.8

Pairwise comparisons indicated that each pairwise difference was significant (p<0.05). Although there was a significant decrease between the knowledge immediately after and 6-months later, there was still a significant increase between the baseline and the evaluation six months later (Table 2).

**Table 2:** Percentage of correct answers in the questionnaire assessing the theoretical knowledge: pairwise comparisons between the three evaluations.

Pairwise comparisons		Mean difference	Standard error	95%CI for difference	
				Lower bound	Upper bound
1	2	-34.515*	0.953	-36.807	-32.224
	3	-23.324*	0.923	-25.545	-21.103
2	1	34.515*	0.953	32.224	36.807
	3	11.191*	0.946	8.917	13.465
3	1	23.324*	0.923	21.103	25.545
	2	-11.191*	0.946	-13.465	-8.917

95%CI: 95% confidence interval.

Based on estimated marginal means.

*The mean difference is significant at the 0.05 level.

1: baseline; 2: one day to one week after training; 3: 6-months after training.

The change in children’s confidence in their own ability to perform CPR when measured before, one day to one week, and 6 months after participation in the training course was also evaluated through a one-way repeated measured ANOVA (n=392). Descriptive statistics are displayed in Table 3. The results indicated a significant time effect (Wilks’ Lambda=0.7, *F(2,342)*=83.3, p<0.05, η^2^=0.3). The Mauchly’s Test of Sphericity indicated that the assumption of sphericity had not been violated, chi-square(2)=4.6, p=0.10.

**Table 3:** Number of points on the questionnaire assessing self-efficacy: descriptive statistics.

	Mean	Standard deviation
Baseline	1.6	1.2
One day to one week after training	2.5	1.3
6-months after training	2.2	1.3

Once again, there was a significant decrease between the self-efficacy immediately after and 6-months after the training course, but there was still a significant increase between the baseline and the evaluation 6-months later (Table 4).

**Table 4:** Number of points on the questionnaire assessing the self-efficacy: pairwise comparisons between the three evaluations.

Pairwise comparisons		Mean difference	Standard error	95%CI for difference	
				Lower bound	Upper bound
1	2	-0.892*	0.070	-1.100	-0.685
	3	-0.596*	0.077	-0.822	-0.370
2	1	0.892*	0.070	0.685	1.100
	3	0.297*	0.077	0.069	0.524
3	1	0.596*	0.077	0.370	0.822
	2	-0.297*	0.077	-0.524	-0.069

95%CI: 95% confidence interval.

Based on estimated marginal means.

*The mean difference is significant at the 0.05 level.

1: baseline; 2: one day to one week after training; 3: 6-months after training.

The classes had, from the start, a baseline knowledge that was statistically different. Therefore, in order to determine whether different classes (with different tutors) had different improvements, two new variables were computed: “*immediately_after - baseline*” and “*6 months_after - baseline*”, which represent the improvement of correct answers, respectively, one day to one week, and six months after the training session, compared to the baseline knowledge. There was a statistically significant difference between the classes in the improvement of correct answers right after the training session (p<0.05). However, there was no statistically significant difference between the classes in the improvement of correct answers at 6 months compared to the baseline knowledge, as determined by one-way ANOVA (p=0.19).

## DISCUSSION

This is the first Portuguese study carried out to evaluate the outcome on theoretical knowledge and self-efficacy of a BLS training session provided by medical students to schoolchildren.

The overwhelming majority of children had no difficulties retaining theoretical knowledge related to performing BLS in the six months period after a single 120-minute training session. As expected, there was a substantial increase immediately after the training session, upholding that the key messages were clearly transmitted by the medical students and correctly understood by the students. Also, the concepts were remembered for the subsequent six months, and despite the lower number of correct answers, there was still a significant difference compared to baseline knowledge. These findings are in line with the concept well established in the “Kids Save Lives” statement,^12^ that children aged 12 years old or less are an appropriate target population for BLS training. Likewise, Bohn et al*.* ^11^ and Connolly et al*.* ^10^ demonstrated that the theoretical knowledge required to perform BLS is within the reach of ten year-old students and can easily be learnt and remembered.

Additionally, the training session improved the schoolchildren’s confidence in their own ability to perform CPR, as described in other studies,^7^ ^,^ ^8^ ^,^ ^11^ indicating the likelihood of a practical application of the knowledge. Once again, the students’ confidence decreased 6 months after the training session but remained substantially higher than the baseline.

The baseline knowledge varied between the 21 classes, which indicate that the overall awareness level regarding this subject is variable among the population. Despite that, the improvement on the number of correct answers was homogeneous, even though each class was taught by a different pair of medical students, suggesting that even with different tutors, it is possible to obtain similar results in terms of acquisition of knowledge on concepts if the support presentation is standardized. As the second questionnaire was answered one day to one week after the presentation, some heterogeneity in the results among the classes on that questionnaire may be due to that time difference.

There are other studies with medical students as CPR trainers and positive results in terms of theoretical knowledge,^10^ meaning that a project that promotes the teaching of BLS by medical students can be an efficient way to increase bystander CPR rates, as they are part of a motivated group and aware of the importance of such programs.

The ideal age for starting CPR training has been subject of a lot of debate.^7^ ^,^ ^8^ ^,^ ^10^ ^,^ ^11^ In this study, the selected target age group of seven to 12 years was based on the Portuguese education system. Elementary schools usually have smaller classes, fewer teachers and shorter curriculums, which made it easier to include the project in the curricular plan. Additionally, despite the discussion concerning the ideal age, the introduction of BLS training shouldn’t be delayed. This project was exceedingly well received by the school community, and almost all elementary schools agreed or even asked to participate in the next edition, planned for November 2017.

There are some limitations in this study. The questionnaire used to valuate the schoolchildren’s theoretical knowledge about CPR lacks a robust validation, but no previously validated questionnaire was identified for use in this study. Additionally, some adaptations to the self-efficacy questionnaire had to be made after realizing that the four-point scale originally used in other studies wasn’t properly understood by the students. The second questionnaire was answered in a period of time that perhaps should have been shorter or at least closer across different classes. However, due to weekends and holidays, it wasn’t possible for some classes to answer it in the day immediately after the training session. Due to the lack of mannequins available, it was impossible to conduct a proper practical assessment of the CPR technic in terms of chest compression depth and compression rate. For that reason, there was no opportunity to determine whether the acquisition of theoretical knowledge and self-efficacy translates into actual CPR skills. We hope that in the future it will be possible to use mannequins to assess CPR practical skills and that the questionnaire can be properly validated, so that a more robust set of results can be evaluated.

Given the children’s excellent performance, this study supports the concept that a single 120-minute BLS training session provided by medical students to schoolchildren is effective in promoting not only the acquisition of theoretical knowledge, but also the confidence in the ability to perform CPR, with results lasting for over a 6-month period. Furthermore, it validates that CPR training can be taught and learnt by the Portuguese schoolchildren, a concept that was already well established in many other studies, from other countries.
